# Investigating Algal Communities in Lacustrine and Hydro-Terrestrial Environments of East Antarctica Using Deep Amplicon Sequencing

**DOI:** 10.3390/microorganisms8040497

**Published:** 2020-03-31

**Authors:** Yuu Hirose, Takuhei Shiozaki, Masahiro Otani, Sakae Kudoh, Satoshi Imura, Toshihiko Eki, Naomi Harada

**Affiliations:** 1 Department of Applied Chemistry and Life Science, Toyohashi University of Technology, Aichi 441-8580, Japan; eki@chem.tut.ac.jp; 2 Earth Surface System Research Center, Japan Agency for Marine-Earth Science and Technology, Kanagawa 237-0061, Japan or takuhei.shiozaki@gmail.com (T.S.); haradan@jamstec.go.jp (N.H.); 3 Current address: Atmosphere and Ocean Research Institute, The University of Tokyo, Chiba 277-8564, Japan; 4 Faculty of Agriculture, Niigata University, Niigata 950-2181, Japan; otani@agr.niigata-u.ac.jp; 5 National Institute of Polar Research, Corporation Research Organization of Information and Systems, Tokyo 190-8518, Japan; kudoh.sakae@gmail.com (S.K.) imura@nipr.ac.jp (S.I.); 6 Department of Polar Science, SOKENDAI (The Graduate University for Advanced Studies), Tokyo 190–8518, Japan

**Keywords:** algae, Antarctica, lacustrine, hydro-terrestrial, Cyanobacteria, microbiome, Tardigrade

## Abstract

Antarctica has one of the most extreme environments on Earth, with low temperatures and low nutrient levels. Antarctica’s organisms live primarily in the coastal, ice-free areas which cover approximately 0.18% of the continent’s surface. Members of Cyanobacteria and eukaryotic algae are important primary producers in Antarctica since they can synthesize organic compounds from carbon dioxide and water using solar energy. However, community structures of photosynthetic algae in Antarctica have not yet been fully explored at molecular level. In this study, we collected diverse algal samples in lacustrine and hydro-terrestrial environments of Langhovde and Skarvsnes, which are two ice-free regions in East Antarctica. We performed deep amplicon sequencing of both 16S ribosomal ribonucleic acid (rRNA) and 18S rRNA genes, and we explored the distribution of sequence variants (SVs) of these genes at single nucleotide difference resolution. SVs of filamentous Cyanobacteria genera, including *Leptolyngbya*, *Pseudanabaena*, *Phormidium*, *Nodosilinea*, *Geitlerinama*, and *Tychonema*, were identified in most of the samples, whereas *Phormidesmis* SVs were distributed in fewer samples. We also detected unicellular, multicellular or heterocyst forming Cyanobacteria strains, but in relatively small abundance. For SVs of eukaryotic algae, Chlorophyta, Cryptophyta, and Ochrophyta were widely distributed among the collected samples. In addition, there was a red colored bloom of eukaryotic alga, *Geminigera cryophile* (Cryptophyta), in the Langhovde coastal area. Eukaryotic SVs of *Acutuncus antarcticus* and/or *Diphascon pingue* of Tardigrada were dominant among most of the samples. Our data revealed the detailed structures of the algal communities in Langhovde and Skarvsnes. This will contribute to our understanding of Antarctic ecosystems and support further research into this subject.

## 1. Introduction

Photosynthesis is the most important biological process that converts solar energy into chemical energy. Members of Cyanobacteria, eukaryotic algae, and plants harbor two types of protein complex, namely photosystem I and II, and synthesize carbohydrate from carbon dioxide and water using light energy with production of oxygen [1]. These photoautotrophic organisms can inhabit environments that are free of organic carbons and serve as primary producers in most environments. Antarctica has one of the most extreme environments on earth, with low temperatures and low nutrient levels, and is perennially and almost entirely covered with thick ice. Living organisms in Antarctica are found primarily in the coastal, ice-free areas, which cover only 0.18% of the continent’s surface [2]. Cyanobacteria usually form microbial mats in the benthos of lakes, ponds, and streams [3]. Diverse eukaryotic algae, such as Chlorophyta (green algae), Bacillariophyta (diatoms), and Xanthophyta (yellow-green algae), have also been reported [4]. They are often exposed to severe physical stresses, such as high irradiation, desiccation, and freeze–thaw cycles [5]. Investigation of their community structures is important to reveal how they have adapted to the stressful environments.

Langhovde and Skarvsnes in Soya Coast, East Antarctica, are the research sites that have long been explored by the Japanese Antarctic Research Expedition. There are more than 50 lakes in these areas [6]. Most of these lakes are oligotrophic, where nutrient concentrations, such as nitrate, nitrite, ammonium, and phosphate, are lower than 0.1 mg l^−1^ [7]. In the bottom of these lakes, where the water does not freeze throughout the year [8], unique tower-like structures of microbial mats, called moss pillars, have been observed [9,10]. Studies using DNA cloning and sequencing of ribonucleic acid (rRNA) genes revealed the detailed community structure of the moss pillars in Lake Hotoke Ike in Skarvsnes [11,12]. The moss pillars consist of *Leptobryum wilsonii* and, in some cases, *Bryum pseudotriquetrum* [9,13]. The most dominant eukaryotic taxon in the moss pillars is Chytridiomycota (Fungi), which may be parasites and/or decomposers [11]. Ciliophora, Labyrinthulomycetes (Sagenista), and Tardigrades were also identified. For prokaryotes, the moss pillars are dominated by Proteobacteria, Cyanobacteria, Chloroflexi, and Planctomycetes [11]. Cyanobacteria genera such as *Leptolyngbya*, *Nostoc*, *Phormidium*, and *Synechococcus* are identified in the exterior part of the moss pillars [11]. Some Cyanobacteria may contribute to nitrogen cycling with their capacity of nitrogen fixation [14]. On the other hand, the community structures in littoral zones of the lakes and hydro-terrestrial environments, which are exposed to severer physical stresses compared with benthic environments, have not been fully explored at a molecular level in Langhovde and Skarvsnes. Recently, community structure of the surface sediment of littoral zone of Lake Yukidori Ike in Skarvsnes was analyzed using the DNA-cloning approach, identifying Proteobacteria, Cyanobacteria, Tardigrada, and Cryptomycoda (Fungi) as dominant taxa [15].

Deep amplicon sequencing of 16S and 18S rRNA genes, using next-generation sequencers, can detect living organisms with a high degree of sensitivity. This approach is replacing traditional analysis methods, such as DNA cloning or denaturing gradient gel electrophoresis (DGGE). The sequences produced by the next generation sequencers, such as the Illumina sequencers, contain ~0.1% sequence errors [16]. Traditionally, these sequences were clustered into operational taxonomic units (OTUs) based on a distance matrix at a specified threshold (e.g., 97% sequence identity), and representative sequences of the OTU were selected [17,18]. However, this approach ignores sequence variations below the identity threshold and causes inflation of minor OTUs containing sequencing errors. Recently, software packages that remove sequence errors have been developed [19,20,21]. These packages produce sequence variants (SVs) that are differentiated at single base-pair resolution, which overcomes the difficulties of the OTU-based approach.

In this study, we analyzed the structures of diverse algal communities in the lacustrine and hydro-terrestrial environments of Langhovde and Skarvsnes using deep amplicon sequencing of both 16S and 18S rRNA genes. We revealed that members of filamentous Cyanobacteria and Tardigrades were widespread in these environments. We also identified other SVs that were less widespread but dominant in specific environments. These results deepen our understanding of the diversity of the lacustrine and hydro-terrestrial ecosystems in East Antarctica.

## 2. Materials and Methods

### 2.1. Sampling Points

**Figure 1:**
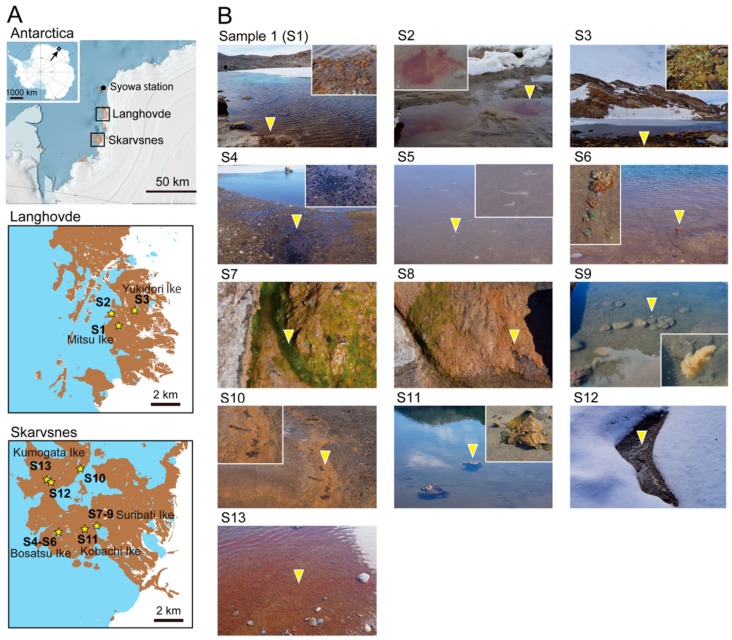
Collection of algal samples. (**A**) Location of sampling sites in Langhovde and Skarvsnes. Sampling sites of S1-S13 were shown as yellow stars. Names of the lakes of the sampling points were shown accordingly. Map data were obtained from Quantarctica package (http://quantarctica.npolar.no/) or Geospatial Information Authority of Japan (https://www.gsi.go.jp/antarctic/) with modifications. (**B**) Photographs of sampling positions of S1-S13 samples. The inlet photographs show the enlargement of the sampling positions indicated by yellow triangles.

Langhovde and Skarvsnes are approximately 25 km and 50 km from Syowa Station on East Ongul Island, respectively (Figure 1A). In these areas, late May to mid-July is the winter season, with polar nights, whereas December to February is the summer season, with white nights. The number of days above 0 °C, recorded by automatic weather stations between 2010 and 2011, were 102 for Langhovde and 74 for Skarvsnes [22]. In the summer period, the average daily temperature was approximately 2 °C in both locations, with average solar radiation of ~200 W/m^2^ [22].

Samples were collected from lacustrine and hydro-terrestrial environments during the summer season, from December 24, 2018 to January 13, 2019, by the Japanese Antarctic Research Expedition. Latitude and longitude of the sampling points were measured using GPSMAP 64s (GARMIN Ltd., Schaffhausen, Switzerland) (Table S1). Samples S1–3 were collected in Langhovde: S1 was a brown mat isolated from Lake Mitsu Ike; S2 was a red colored bloom observed in a puddle of thawing snow in the coastal area; and S3 was a green and brown mat that was collected in Lake Yukidori Ike (Figure 1B). The S4–13 samples were collected in Skarvsnes: samples S4–6 were collected in Lake Bosatsu Ike (S4 and S6 were black and brown bacterial mats, respectively, and S5 was a small white filamentous aggregate floating on the surface of Lake Bosatsu Ike (Figure 1B)); samples S7–9 were collected near Lake Suribati Ike (S7 was a green colored aggregate found in a small stream near Lake Suribati Ike, S8 was a brown mat found near the stream, and S9 was a white aggregate floating on the surface of Lake Suribati Ike (Figure 1B)); S10 was a black and brown mat collected from Lake Neko Ike; S11 was a floating brown mat that originated from the benthic algal mat in Lake Kobachi Ike; S12 was a dark yellow mat collected from thawed soil near Lake Tokkuri Ike; and S13 was a orange mat, found at the bottom of the shallows in Lake Kumogata Ike. S2, S5, S7, and S9 were collected with liquid water, whereas the others were wet mat or soil samples. The pH and salinity of water samples were recorded using a portable analyzer PC5 (CEM Corporation, Bunkyo-ku, Tokyo, Japan). The water pH at these sampling points ranged from pH 7.03 to 8.87, and the salinity ranged from 0.02 to 7.5 parts per thousand (ppt) (Table S1).

### 2.2. Sample DNA Preparation

Approximately 10–30 g of samples were collected from 1–2 cm of the surface of each microbial mat, aggregate or soil, using a scoop. S2 water sample was collected using a disposable plastic syringe. Samples were transferred to the icebreaker SHIRASE and then frozen until DNA extraction was performed. Genomic DNA was extracted from approximately 10 mL volumes of each sample. The samples were mixed with 10 mL of 10 mM Tris-HCl pH 8.0, 5 mL of phenol pH 8.0, and 2 g of zirconia/glass beads (ϕ 0.1 mm), and vortexed vigorously for three minutes at room temperature. After heat treatment at 65 °C for 10 min, the debris and beads were removed by centrifugation for five minutes at 16,000× *g*. The upper water phase was transferred to a new tube and an equal volume of chloroform/isoamylalchol (24:1) was added, vortexed vigorously, and centrifuged for five minutes at 16,000× *g*. The upper water phase was precipitated with 2.5 volumes of 99.5% ethanol and 0.1 volumes of sodium acetate pH 5.2, and precipitated with centrifugation for 10 min at 21,600× *g*. The white pellets containing genomic DNA were washed with 70% ethanol, dried for five minutes at room temperature, and dissolved with 300 μl of 10 mM Tris-HCl pH 8.5. DNA was further purified using a DNeasy Blood and Tissue kit (Qiagen, Hilden, Germany), in accordance with the manufacturer’s instructions, and eluted with water.

### 2.3. Library Preparation and Sequencing

For deep amplicon sequencing, the V3–V4 region of the 16S rRNA and the V7–V8 region of the 18 rRNA genes were amplified using KOD FX Neo (Toyobo, Osaka, Japan). Primer sets 341F and 805R were used for 16S rRNA analysis [23], whereas primer sets F1183 and R1631 were used for 18S rRNA analysis [24]. The nucleotide sequences of these primers including the Illumina adapter for indexing were as follows (annealing sequences are underlined):

341F: 5′- TCGTCGGCAGCGTCAGATGTGTATAAGAGACAGCCTACGGGNGGCWGCAG-3′;

805R: 5′-GTCTCGTGGGCTCGGAGATGTGTATAAGAGACAGGACTACHVGGGTATCTAATCC -3′;

F1183: 5′- TCGTCGGCAGCGTCAGATGTGTATAAGAGACAGAATTTGACTCAACACGGG-3′;

R1631: 5′- GTCTCGTGGGCTCGGAGATGTGTATAAGAGACAGTACAAAGGGCAGGGACG-3′.

The polymerase chain reaction (PCR) thermal cycle was an initial denaturing step at 94 °C for two minutes, 35 cycles of denaturation at 98 °C for 10 s, annealing at 55 °C for 30 s, and extension at 68 °C for 30 s, with the final extension step at 68 °C for five minutes. The PCR product was purified with 0.8 volumes of AMPure XP beads, in accordance with the manufacturer’s instructions, and eluted with 10 mM Tris-HCl pH 8.5. Index PCR was performed in eight cycles using a Nextera XT Index Kit v2 (Illumina, San Diego, California, USA), in accordance with the manufacturer’s instructions. The same index was used for the 16S and 18S rRNA amplicons that were obtained from the same sample. The amplified libraries were purified by the addition of 1.12 volumes of AMPure XP beads, in accordance with the manufacturer’s instructions, and eluted with 10 mM Tris-HCl pH 8.5. The concentration of each library was quantified using a spectrophotometer, and equal amounts of each library were pooled and quantified using a Qubit dsDNA HS Assay Kit (Thermo Fisher Scientific, Waltham, Massachusetts, USA). Each 300 bp end of the pooled library was sequenced using an MiSeq Reagent Kit v3 (600 cycles; Illumina) on the MiSeq instrument (Illumina). The sequences were deposited at the DDBJ Sequence Read Archive (DRA) database under the accession numbers DRR205705 to DRR205717 with BioProject ID PRJDB9246 and BioSample IDs SAMD00202801 to SAMD00202813.

### 2.4. Data Analysis

Several noise removal software packages, such as DADA2 [19], Deblur [20], and UNOISE3 [21], have recently become available. These packages produce denoised sequences called amplicon sequence variants [19], sub-OTUs [20], or zero-radius OTUs [21]. These terms are synonymous, and are referred to as “sequence variants” (SVs) in this study. The three denoising packages produce a similar composition of SVs, but a different number of SVs [25]. DADA2 demonstrated the highest sensitivity to finding lower-abundance SVs, with false-positives, whereas Deblur had fewer abundant SVs, but a lower number of false positives [25]. We utilized the conservative Deblur package in this study. The sequence reads of the 16S and 18S rRNA genes were split using Cutadapt v1.8.3 [26]. We used the -g ^CCTACGGGNGGCWGCAG and -G ^GACTACHVGGGTATCTAATCC options for 16S rRNA analysis and the -g ^AATTTGACTCAACACGGG -G ^TACAAAGGGCAGGGACG options for 18S rRNA analysis. Additionally, we used the --discard-untrimmed option to eliminate sequence reads that did not contain the 5′ anchored adaptors. The sequence reads were imported QIIME2 ver. 2019.10 (https://qiime2.org) [27]. Forward and reverse reads were joined, denoised and chimera checked using the Deblur plugin with --p-trim-length options of 400 for both 16S rRNA and 18S rRNA genes. The taxonomy of the SVs was assigned using a feature-classifier plugin that was trained with the taxonomy information in majority_taxonomy_7_levels.txt of 99% clustering in SILVA ver. 132 (https://www.arb-silva.de/download/archive/) [28], for 16S rRNA analysis, or pr2_version_4.12.0_18S_mothur.tax of the Protist Ribosomal Reference database (PR2) [29], for 18S rRNA analysis. For 16S rRNA analysis, the SVs derived from eukaryotic chloroplasts and mitochondria were not removed. Some chimeric sequences were retained, even after the de novo chimera check pipeline of Deblur, which was probably caused by DNA fragmentation after our glass/zirconia beads treatment. Therefore, we performed a reference-based chimera check using vsearch with the --minh 0.5 option, using SILVA_132_SSURef_tax_silva.fasta as a reference [30], which removed 308 chimeric SVs (9.6%) for the 16S rRNA and 43 chimeric SVs (5.8%) for the 18S rRNA. Finally, 2,824 SVs and 692 SVs were obtained for the 16S and 18S rRNA genes respectively (S1 and S2 Data). To distinguish between the SV IDs of the 16S and 18S rRNA genes, they were designated as 16SV_XX and 18SV_XX, respectively (S1 and S2 Data). The number of ID was assigned in descending order of the sums of the counts of each SV. For phylogenetic tree construction, 92 SVs assigned to Cyanobacteria phylum, not belonging to chloroplast, were obtained using the filter_taxa function of the R phyloseq package [31]. Multiple sequence alignment of these SVs was prepared using the SILVA Incremental Aligner (SINA v1.2.11) with reference alignment of SILVA_132_SSURef_NR99_13_12_17_opt.arb [32]. The phylogenetic tree was estimated using FastTree v2.1.7 [33] and visualized using iTol v4 [34]. The relative abundance of major SVs were illustrated on bar and balloon plots (phyloseq v1.28.0, ggplot2 v3.2.1) by selecting SVs representing at least 0.2% of the overall dataset [31,35]. For α and β diversity analyses, SVs were rarefied to an even depth of 11,986 and 8,772 sequences, for 16S and 18S rRNA genes, respectively, using the R phyloseq package’s rarefy_even_depth function [31]. Non–metric multidimensional scaling (NMDS) plot of the Bray–Curtis distance matrix was obtained using ordinate and plot_ordination functions in the R phyloseq package [31]. Local alignment of SVs against the GenBank database and sequence identity analysis were performed using the blastn program [36].

## 3. Results and Discussion

### 3.1. Overall Community Structures

**Figure 2:**
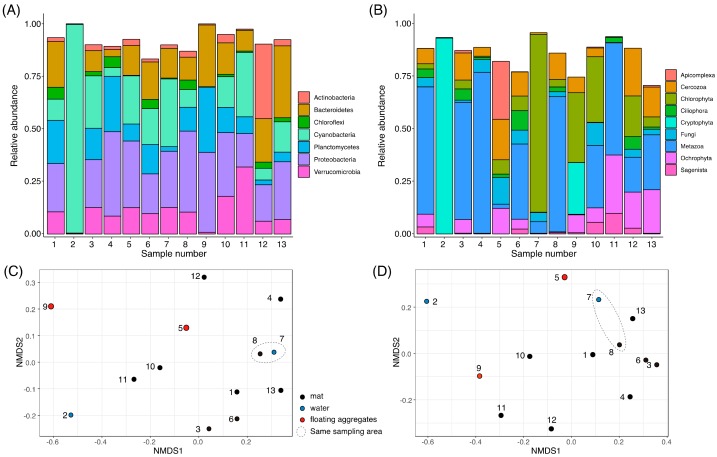
Overall community structures of collected samples. Bar plots show the composition of sequence variants (SVs) that were agglomerated to the phylum level classification of the SILVA ver. 132 database for 16S rRNA (**A**) or the Rank 3 classification of the Protist Ribosomal Reference (PR2) database for 18S rRNA (**B**). Taxa below an average frequency of 2% are not shown. The NMDS plots of the Bray–Curtis distance matrix of each sample are shown for 16S rRNA (**C**) and 18S rRNA (**D**).

We analyzed community structures of diverse algal samples (S1–S13) that were collected from lacustrine and hydro-terrestrial environments of Langhovde and Skarvsnes (Figure 1, Table S1). Bar plots of the phylum level classification of the 16S rRNA showed that the dominant taxa were Bacteroidetes, Cyanobacteria, Planctomycetes, Proteobacteria, and Verrucomicrobia (Figure 2A). Previous study showed that Proteobacteria, Cyanobacteria, Chloroflexi, and Planctomycetes were dominant in the external parts of moss pillars [11]. Chloroflexi was detected in all our samples, but its relative abundance was low compared with the other dominant phyla (Figure 2A). Verrucomicrobia, which is nearly ubiquitous in soil environments including Antarctica [37], was detected in most samples, but in low abundances in S2 and S9 (Figure 2A).

The relative abundance of Cyanobacteria was high in S2 (99.2%), S7 (32.2%), S11 (30.7%), S3 (24.9%), and S5 (23.0%), when compared with S6 (17.1%), S10 (14.6%), S13 (14.4%), S1 (10.1%), S8 (8.4%), S12 (5.6%), S4 (4.1%), and S9 (0.5%) (Figure 2A). The domination of Cyanobacteria in S2 is caused by chloroplast sequence of Cryptophyta forming red bloom (discussed in 3.4.). The orange colored Cyanobacteria mats like S1, S3, S6, S11, and S13 (Figure 1) were often observed in benthic environments of Antarctica, which is caused by accumulation of photo-protective pigments, such as carotenoids, scytonemin, and mycosporine-like amino acids [38]. These Cyanobacterial pigments may also protect other living organisms from UV irradiation. The abundance of Actinobacteria was high in S12 (35.5%), but not in other samples (Figure 2A). The abundances of Actinobacteria and Cyanobacteria showed negative and positive correlation, respectively, with moisture availability in the hyporheic zone of McMurdo Dry Valleys, Antarctica [39]. The high Actinobacteria and low Cyanobacteria abundances in S12 may suggest that the soil environment around S12 could be exposed to the low moisture condition.

The NMDS plot of the Bray–Curtis dissimilarity suggested a similar community structure in S7 and S8 (Figure 2C), which were collected from the same sampling point (Table S1). The NMDS plot suggested that the community structures of S2 and S9 tend to be distantly related to other samples (Figure 2C). The S2 showed the lowest alpha diversity (identified SVs, Shannon index, and Simpson index) at the rarefied read depth (Figure S1).

For 18S rRNA analysis, bar plots of the rank 3 (division) classification showed that the dominant taxa are Chlorophyta, Ochrophyta, Cryptophyta, Cercozoa and/or Metazoa, whose compositions are substantially different between samples (Figure 2B). The relative abundance of Chlorophyta was high in S7 (88.0%), S9 (34.5%), S10 (32.4%), and S12 (20.2%), whereas that of Ochrophyta was high in S11 (28.9%), S13 (21.6%), and S12 (17.9%). The relative abundance of Cryptophyta was high in S2 (96.9%) and S9 (25.7%), very low in S10 (0.23%), and not detected in the other samples. In addition to algae, Metazoa was widespread in S4 (79.8%), S8 (66.9%), S1 (63.0%), S3 (58.1%), S11 (55.6%), S6 (37.2%), S10 (30.9%), S13 (27.2%), and S7 (5.8%), and represented less than 3% in the other four samples. In the community structure of moss pillars, over half of eukaryotic phylotypes were assigned to Fungi [12], but Fungal SVs were not dominant in all our samples.

### 3.2. Distribution of SVs with High Prevalence

**Figure 3:**
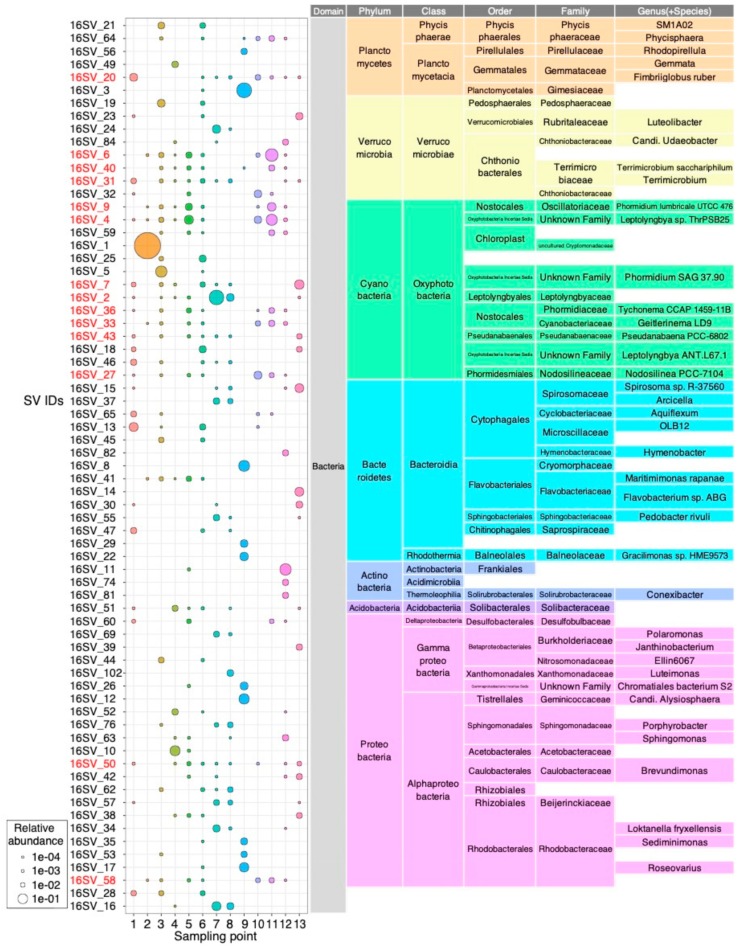
Relative abundance and assigned taxonomy of the major SVs (16S rRNA gene) retrieved from 13 lacustrine and hydro-terrestrial samples collected in Langhovde and Skarvsnes, East Antarctica. SVs had been represented if they reached a relative abundance over 0.2% of the overall dataset. IDs of SVs with high prevalence (>6/13 samples) are shown in red.

In 16S rRNA analysis, we identified 69 major SVs (Figure 3): 21 SVs of Proteobacteria; 14 SVs of Bacteroidetes; 14 SVs of Cyanobacteria; eight SVs of Verrucomicrobia; six SVs of Planctomycetes; and six other SVs. Twelve SVs were detected in only one sample, and the other 57 SVs were detected multiple times in different samples. In particular, 14 SVs showed a high prevalence in over half of the samples (>6/13) (red colored SVs in Figure 3). Eight of these SVs belonged to the Cyanobacteria phylum, which contained *Phormidium* (16SV_9 and 16SV_7), *Leptolyngbya* (16SV_4), *Tychonema* (16SV_36), *Geitlerinema* (16SV_33), *Pseudanabaena* (16SV_43), *Nodosiliea* (16SV_27), and the Leptolyngbyaceae family (16SV_2). These SVs showed 100% sequence identity to rRNA sequences in GenBank database that were detected in other Antarctic environments. For example, *Phormidium* (16SV_9) corresponds to *Phormidium pseudopriestleyi* FRX01 (KT347094) isolated in McMurdo Dry Valley, Southern Victorialand [40]. *Phormidium* (16SV_7) corresponds to the uncultured Cyanobacteria clone derived from Larsemann Hills (JX172507) [41]. *Leptolyngbya* (16SV_4) corresponds to *Leptolyngbya antarctica* ANT.BFI.1 isolated from Firelight Lake, Larsemann Hills (AY493590) [42]. *Nodosiliea* (16SV_27) corresponds to *Leptolyngbya antarctica* ANT.LAC.1 isolated from Ace Lake Peninsula, Vestfold Hills [42]. Leptolyngbyaceae (16SV_2) showed a 99.47% sequence identity to *Phormidesmis arctica* HOR 11-6 (KU219729.1) isolated from Svalbard in the Arctic. It also showed a 99.75% identity to Oscillatoriales OTU7 that were found in cryoconites on the glaciers of Antarctica, Arctic, Green land, and Northern Asia [43]. In addition to Cyanobacteria, six SVs showed a high prevalence (red colored SVs in Figure 3): *Fimbriiglobus ruber* (Planctomycetes: 16SV_20), Chthoniobacterales (Verrucomicrobia: 16SV_6, 16SV_40, and 16SV_31), *Brevundimonas* (Proteobacteria: 16SV_50)*,* and *Roseovarius* (Proteobacteria: 16SV_58).

**Figure 4:**
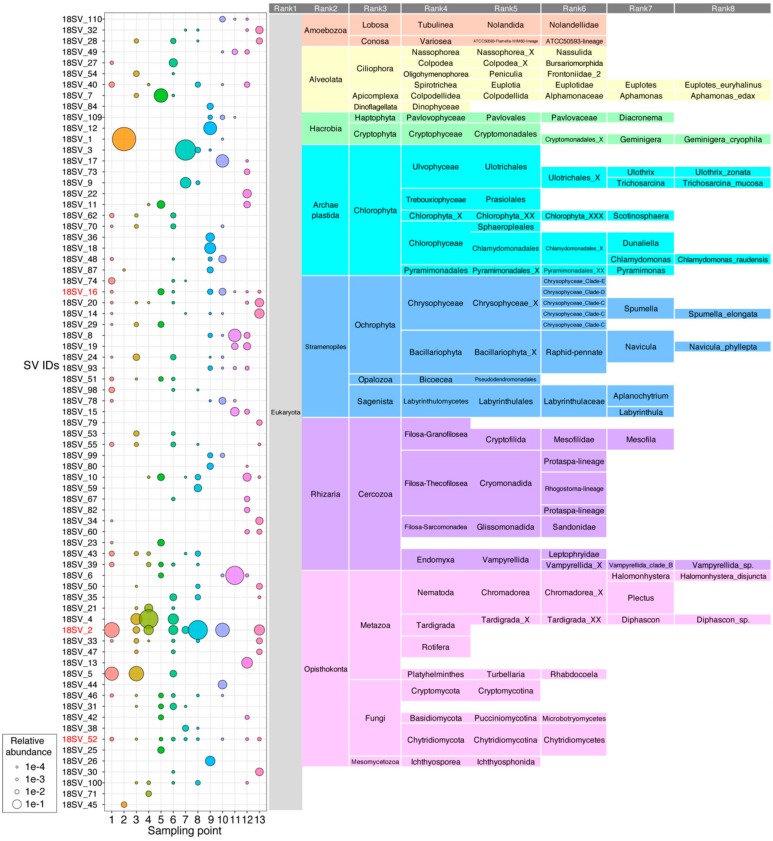
Relative abundance and assigned taxonomy of the major SVs (18S rRNA gene) retrieved from 13 lacustrine and hydro-terrestrial samples collected in Langhovde and Skarvsnes, East Antarctica. SVs had been represented if they reached a relative abundance over 0.2% of the overall dataset. IDs of SVs with high prevalence (>6/13 samples) are shown in red. Unidentified ranks were shown as ‘_X’ in as described in the PR2 database [29].

In 18S rRNA analysis, we identified 73 major SVs (Figure 4). Thirteen SVs were detected in only one sample, and the other 60 SVs were detected multiple times in different samples. The prevalence of the major SVs in the 18S rRNA communities tended to be lower than that of the 16S rRNA communities. For example, only three SVs were detected in over half the samples (>6/13) (red colored SVs in Figure 4), which contained Chrysophyceae (Ochrophyta: 18SV_16), Tardigrada (Metazoa: 18SV_2), and Chrytridiomycetes (Fungi: 18SV_52). Chrysophyceae (18SV_16) showed a 100% sequence identity to *Ochromonas* sp. CCMP1899 isolated from McMurdo Sound, Antarctica (EF165133). Chrytridiomycetes (18SV_52) showed a 100% identity to uncultured Rhizophydiales in seawater of Japan (AB971109). Distribution of Tardigrada were discussed in 3.7.

### 3.3. Distribution of Cyanobacteria SVs

**Figure 5:**
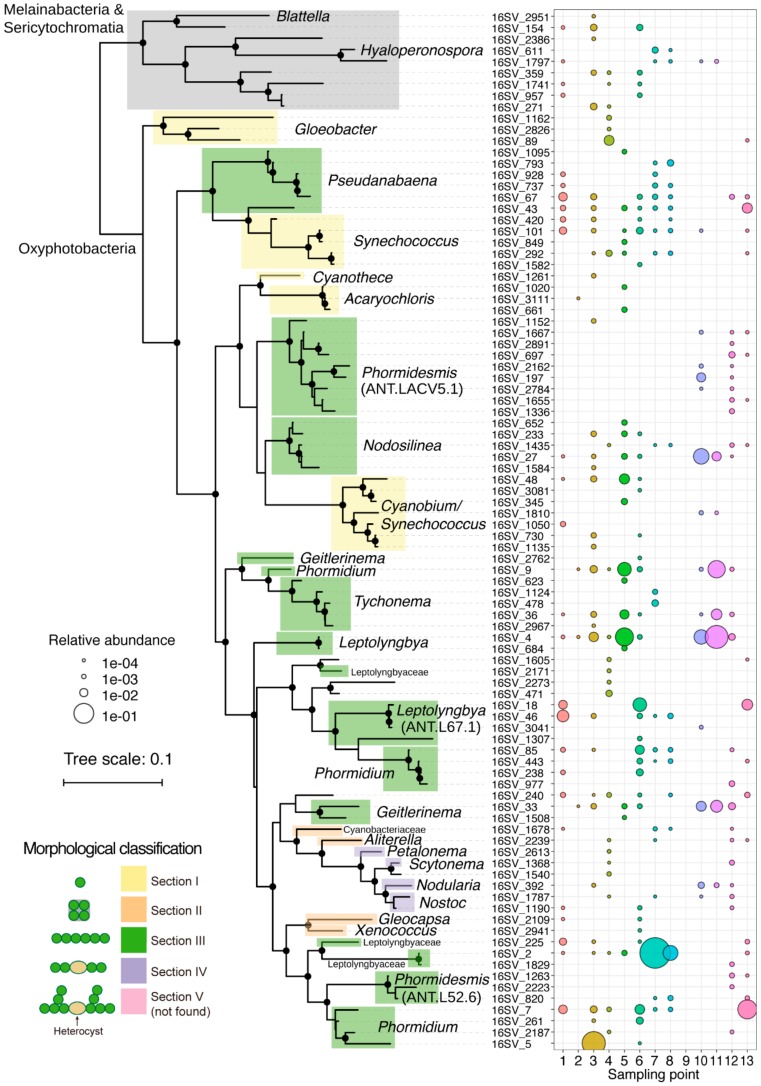
Phylogenetic tree, relative abundance, and genus level taxonomy of all 92 SVs of cyanobacteria other than chloroplast detected. Branches with a confidence value >80% in the Shimodaira-Hasegawa (SH) test using the FastTree program are shown as black circles [33]. Morphological classifications of sections I-V in Cyanobacteria were shown accordingly.

We focused on all detected SVs of Cyanobacteria phylum other than chloroplast (total 92 SVs), and we plotted their distribution and their phylogenetic relationship (Figure 5). Thirty-six SVs were detected in only one sample, and the other 56 SVs were detected multiple times in different samples (Figure 5). Cyanobacteria phylum are classified into Oxyphotobacteria, Melainabacteria, and Sericytochromatia [44,45], where the ability of oxygen-evolving photosynthesis has emerged only among Oxyphotobacteria. Oxyphotobacteria have traditionally been classified into five sections, based on their morphology [46]: (I) unicellular; (II) multicellular; (III) non-branched filamentous; (IV) non-branched filamentous and capable of forming differentiated cells for nitrogen fixation (heterocystous) [47]; and (V) blanched filamentous and heterocystous. Phylogenetic study showed that most extant Cyanobacteria descend from multicellular ancestors [48]. Notably, section III SVs were dominant in all samples (Figure 5, green). In contrast, section I SVs (yellow), such as *Gloeobacter*, *Synechococcus*, and *Acaryochloris*, and Section II SVs (orange), such as *Gloeocapsa* and *Xenococcus,* were detected, but in low abundance (Figure 5, yellow). Section IV SVs (purple), such as *Nostoc*, *Nodularia*, *Petalonema*, and *Scytonema*, were detected, but there were only five SVs, and their relative abundances were low. No section V SVs were detected in any samples, even at a sequencing depth over 11,986 sequences/sample (Figure 5 and Figure S2). The low abundance of the heterocystous strains of section IV and V is probably not caused by inefficient DNA extraction, since we utilized physical cell lysis with zirconia/glass beads.

The domination of section III strains was also reported in other studies of benthic microbial mats across Antarctica [14,49,50]. The low abundance of heterocystous strains suggests that nitrogen fixation of our samples was mainly performed by non-heterocystous strains and/or other heterotrophic bacteria. On the other hand, Cyanobacterial mats dominated with heterocystous strains such as *Nostoc commune* were observed in soils and ponds of Antarctica [37,51,52]. *Nostoc* was absent in salt pond with high conductivity on the McMurdo Ice Shelf, Antarctica, where *Nodularia* was still present [53]. The salinity of water in our sampling area ranged from 0.02 to 7.5 ppt (Table S1). Thus, high salt concentration may not inhibit the distribution of section V strains in our samples. Further analyses of community structures and environmental parameters (e.g., moisture, light, nutrient, metals, and oxygen concentration) are required to reveal the different adaptive strategies between heterocystous and non-heterocystous strains in Antarctica. For section III strains, *Leptolyngbya*, *Pseudanabaena*, *Phormidium*, *Nodosilinea*, *Geitlerinama*, and *Tychonema* were widespread in most samples, whereas *Phormidesmis* was distributed in limited number of samples (Figure 5). Eight SVs belonging to *Phormidesmis* ANT. LACV5.1 clade were detected in S12, where the soils were mostly covered with snow (Figure 1B). This distribution suggests that *Phormidesmis* strains of the eight SVs share common molecular mechanism(s) to acclimate to the soils exposed to freeze-thaw cycles. *Phormidesmis* genus is generally cold tolerant and widely distributed in Antarctic, Arctic, and Alpine environments [54,55]. Recent genome analysis of *Phormidesmis priestleyi* BC1401, which was isolated from cryoconite of Greenland, suggested that extracellular polymeric substances (EPS) could contribute to the tolerance of this strain in cold stress [56]. Analyses of the EPS-producing mechanism of *Phormidesmis* and its regulation responding to various physical stresses are important topics to be studied.

### 3.4. Red algal Bloom in S2

There was a brilliant red colored algal bloom in S2 in a puddle of thawing snow (Figure 1B). Such blooms are called red snow and are commonly observed in polar and alpine regions [57]. Cosmopolitan phylotypes of snow algae have been reported across the Arctic and Antarctic [49], suggesting their ability to spread widely. The red colored pigments are carotenoids, such as astaxanthin, that protect cells from excessive ultraviolet and visible light irradiation [58]. 16SV_1 was found in high abundance only in S2 (99.1%) and this SV was assigned to the Cryptomonadaceae chloroplast sequence. 16SV_1 had the highest identity (99.75%), with the chloroplast sequences of *Teleaulax amphioxeia* strain HACCP-CR01 (KP899713.1), *Dinophysis caudata* strain DC-LOHABE01 (EU123324.1), *Myrionecta rubra* strain MR-MAL01 (EU123322.1), and *Dinophysis acuminata* (AB073114.1), and a 99.5% identity to the chloroplast sequence of *Geminigera cryophile* (AB073111.1). The 18SV_1 gene was most abundant in S2 only (96.7%) and was assigned to *Geminigera cryophila*. 18SV_1 showed a 100% identity with *Geminigera cryophila* strain Ace Lake (HQ111513.1), which was detected in Antarctica’s Ace Lake, Vestfold Hills, East Antarctica [59]. The composition of red colored algal blooms in the Yatude Valley, Langhovde, has been reported previously [60]. DGGE analysis showed that these blooms contained *Chlamydomonas* and *Chlorella* (Chlorophyta), which accumulate large amounts of astaxanthin and its derivatives in the cells [60]. Our study showed that tThe red blooms in S2 consisted of only a single strain of *Geminigera cryophile* and contained hardly any other algae (Figure 3 and Figure 4), suggesting that the red blooms’ composition could differ greatly between sampling points in Langhovde. Previous nitrogen isotope analyses suggested that the red snow’s primary nitrogen source was fecal pellets from seabirds [60]. Although we did not identify dominant SVs that are specific to animal gut microbiome in S2 (Figure 3), the microbiome of the fecal pellets of seabirds and its comparison to that of the algal blooms will be an interesting topic to explore in the future.

### 3.5. Dominant Algae Strains in S7 and S8

S7 was green colored aggregates collected from a small stream, whereas S8 was a bacterial mat collected near S7 (Figure 1B). Three major algal SVs were detected in S7: 16SV_2 of Leptolyngbyaceae (26.5%), 18SV_3 (71.3%), and 18SV_9 (16.0%). The 18SV_3 was assigned as an order of Ulotrichales, but it had a 100% identity to the taxa of *Ulvophyceae* (KY233156.1), *Klebsormidium* (JQ315653.1), *Ulothrix* (DQ821516.1), *Chlorothrix* sp. (AY476827.1), and *Urospora* (AY476821.1). The 18SV_9 was assigned as *Trichosarcina mucosa* (Figure 4), but it had a 100% identity to the genera of *Tupiella* (MH374178.1), *Sarcinofilum* (MF000566.1), *Hazenia* (MF000563.1), *Neoclonium* (MF034643.1), *Chamaetrichon* (MF034633.1), *Pseudendoclonium* (DQ011230.1), and *Trichosarcina* (AM109906.1). Therefore, the genus level classifications of 18SV_3 and 18SV_9 remain ambiguous. Other DNA markers, such as the internal transcribed spacer (ITS), are required for accurate taxonomic assignment of 18SV_3 and 18SV_9 SVs. We did not identify chloroplast sequences for the two Ulotrichales, suggesting that the primer sets in the V3–V4 region did not work for the chloroplast DNA of these eukaryotic algae. Thus, the major populations of green colored aggregates observed in S7 were one strain of Leptolyngbyaceae and two strains of Ulotrichales. S8 also contained these three algal SVs, but with smaller relative abundance.

### 3.6. White Aggregates in S5 and S9

We collected two samples of white aggregates: small, filamentous aggregates in S5 and a large aggregate in S9 (Figure 1B). S5 contained filamentous Cyanobacteria, such as 16SV_4 (8.1%) of *Leptolyngbya* and 16SV_9 (4.1%) of *Phormidium*, and some SVs of unicellular Cyanobacteria, such as *Acaryochloris*, *Cyanobium*, and *Synechococcus* (Figure 3 and Figure 5). S5 also contained eukaryotic algal SVs of 18SV_11 (7.1%) of Prasiolales, and 18SV_16 (3.9%) and 18SV_51 (3.4%) of Chrysophyceae. However, these algae were not the majority in S5 (Figure 2A ,B), suggesting that the white aggregate in S5 consists of diverse non-algal species. S5 contained 18SV_7 with high abundance (28.7%), which was assigned as *Alphamonas edax* of Apicomplexa (Figure 4). The 18SV_7 gene had a 98.75% sequence identity to *Colpodella* (formerly *Alphamonas*) *edax* (AY234843.1). This organism, and other minor organisms, may contribute to white aggregate formation in S5. In contrast, S9 consisted of mainly eukaryotic algal SVs of 18SV_12 (25.7%) of Cryptomonadales, and 18SV_18 (17.6%) and 18SV_36 (9.3%) of *Dunaliella* (Figure 4). S9 did not contain any Cyanobacterial SVs other than chloroplasts (Figure 5). These results suggest that the large white aggregate in S9 was a eukaryotic algal mat. Notably, number of SVs, Shannon index, and Simpson index were low in the 16S rRNA community of S9 (Figure S1). It might be possible that the eukaryotic algae in S9 have been exposed to excessive light or high temperature stress on the lake surface and bleached their pigmentation.

### 3.7. Other Dominant SVs of Small Animals

The most dominant eukaryotic SVs were 18SV_2 and 18SV_4, which belonged to the phylum Tardigrada (Figure 4). Tardigrades can survive the severe climate of Antarctica, such as freeze-thaw cycles and dry conditions, by stopping all metabolic processes, which is known as cryptobiosis [61]. The 18SV_2 was identified in S1 (35.4%), S3 (4.6%), S4 (12.0%), S6 (10.8%), S7 (5.5%), S8 (61.8%), S10 (29.2%), and S13 (14.5%), whereas 18SV_4 was identified in S3 (17.5%), S4 (59.3%), and S6 (14.9%). These SVs of Tardigrada were not detected in surface water samples, such as S2, S5, and S9, suggesting that they inhabit benthic and hydro-terrestrial environments rather than open water environments. 18SV_2 had a 100% sequence identity to *Acutuncus antarcticus* (AB753790.1), whereas 18SV_4 had a 100% identity to *Diphascon pingue* (MH079473.1). These two SVs only differed in 3 nucleotides. These results suggest that *Acutuncus antarcticus* was the most widespread at our sampling points in Langhovde and Skarvsnes, whereas *Diphascon pingue* was less widespread, but more dominant, than *Acutuncus antarcticus*. This assumption is consistent with the observation that *Acutuncus antarcticus* is the most common species in terrestrial and lake environments in Antarctica [62]. Recently, the community structure of Lake Yukidori Ike, where the S3 sample was isolated, was analyzed using DNA-cloning methods for 16S and 18S rRNA genes [15]. This study showed that the majority of eukaryotic OTUs were Tardigrada, where OTUs of *Diphascon* (44%) and *Acutuncus* (9%) were detected. The high abundance of *Diphascon* compared with *Acutuncus* in Lake Yukidori Ike was consistent with the result of S3 in our study.

18SV_5 was identified in S3 (27.0%), S4 (33.3%), and S6 (4.5%). 18SV_5 was assigned to the order Rhabdocoela of flatworms (Figure 4). Rhabdocoela (18SV_5) had a 100% sequence identity to the minor phylotype (AB695468.1) in the moss pillars [12] and only a 94.5% sequence identity to *Mesostoma lingua* (AY775759.1). 18SV_6 dominated, especially in S11 (55.6%), and was assigned as *Halomonhystera disjuncta* of Nematode (Figure 4). Consistently, 18SV_6 showed a 100% sequence identity to *Halomonhystera disjuncta* (AJ966485.1) [63]. *Halomonhystera* is a cosmopolitan genus that has been recovered from various marine environments [64]. Stable isotope experiment suggested that *Halomonhystera* utilizes phytoplankton-derived hydrocarbons as energy source in the ice-shelf of the Eastern Antarctic Peninsula [65]. Therefore, we speculate that the *Halomonhystera* (18SV_6) may utilize Cyanobacteria and Ochrophyta as energy sources, as they were abundant in the microbial mat of S11. This information will contribute to our understanding of the diversity and adaptability of the lacustrine and hydro-terrestrial ecosystems in Antarctica.

## Supplementary Materials

Supplementary materials can be found at https://www.mdpi.com/2076-2607/8/4/497/s1.
