# Supplementary material for: Investigating Algal Communities in Lacustrine and Hydro-Terrestrial Environments of East Antarctica Using Deep Amplicon Sequencing
Source: Microorganisms. 2020 Mar 31;8(4):497. doi: 10.3390/microorganisms8040497 (PMC7232531; doi:10.3390/microorganisms8040497)
Supplement: Supplementary file 1 [file microorganisms-08-00497-s001.zip › supporting information/Supporting Information.docx]

**Supporting information**

**S1 Table: Information on the sampling points.** Salinity (Sal) and pH values of S8, S10 and S12 were not available (NA) due to their low water content. Tentative lake name was indicated by asterisk.

| Sample | Date | Latitude | Longitude | Area | Location  (*tentative name) | Environment type | Sample type | Sal (ppt) | pH |
| --- | --- | --- | --- | --- | --- | --- | --- | --- | --- |
| S1 | 2018/12/24 | -69.25 | 39.728 | Langhovde | Lake Mitsu Ike | Lacustrine | mat | 0.49 | 8.59 |
| S2 | 2018/12/31 | -69.2418555 | 39.7162789 | Langhovde | Lake Snow puddle | Hydro-terrestrial | water | 7.5 | 8.87 |
| S3 | 2019/1/4 | -69.2405741 | 39.7564721 | Langhovde | Lake Yukidori Ike | Lacustrine | mat | 0.02 | 7.41 |
| S4 | 2019/1/4 | -69.4770973 | 39.571498 | Skarvsnes | Lake Bosatsu Ike | Lacustrine | mat | 0.18 | 8.3 |
| S5 | 2019/1/4 | -69.4770973 | 39.570983 | Skarvsnes | Lake Bosatsu Ike | Lacustrine | Floating  aggregate | 0.18 | 8.3 |
| S6 | 2019/1/4 | -69.4772177 | 39.5717125 | Skarvsnes | Lake Bosatsu Ike | Lacustrine | mat | 0.18 | 8.3 |
| S7 | 2019/1/5 | -69.48226667 | 39.66231667 | Skarvsnes | near Lake Suribati Ike | Hydro-terrestrial | water | 0.38 | 8.35 |
| S8 | 2019/1/5 | -69.48226667 | 39.66231667 | Skarvsnes | near Lake Suribati Ike | Hydro-terrestrial | mat | NA | NA |
| S9 | 2019/1/5 | -69.4827067 | 39.663 | Skarvsnes | Lake Suribati Ike | Lacustrine | Floating  aggregate | 4.61 | 7.03 |
| S10 | 2019/1/8 | -69.447417 | 39.634728 | Skarvsnes | near Lake Neko Ike* | Hydro-terrestrial | mat | NA | NA |
| S11 | 2019/1/9 | -69.4836089 | 39.6430818 | Skarvsnes | Lake Kobachi Ike | Lacustrine | mat | 1.59 | 7.97 |
| S12 | 2019/1/13 | -69.455 | 39.583 | Skarvsnes | near Lake Tokkuri Ike | Hydro-terrestrial | mat | NA | NA |
| S13 | 2019/1/13 | -69.454 | 39.5787854 | Skarvsnes | Lake Kumogata Ike | Lacustrine | mat | 0.48 | 7.55 |

**S1 Data**

Sequences of SVs of 16S rRNA amplicons.

**S2 Data**

Sequences of SVs of 18S rRNA amplicons.


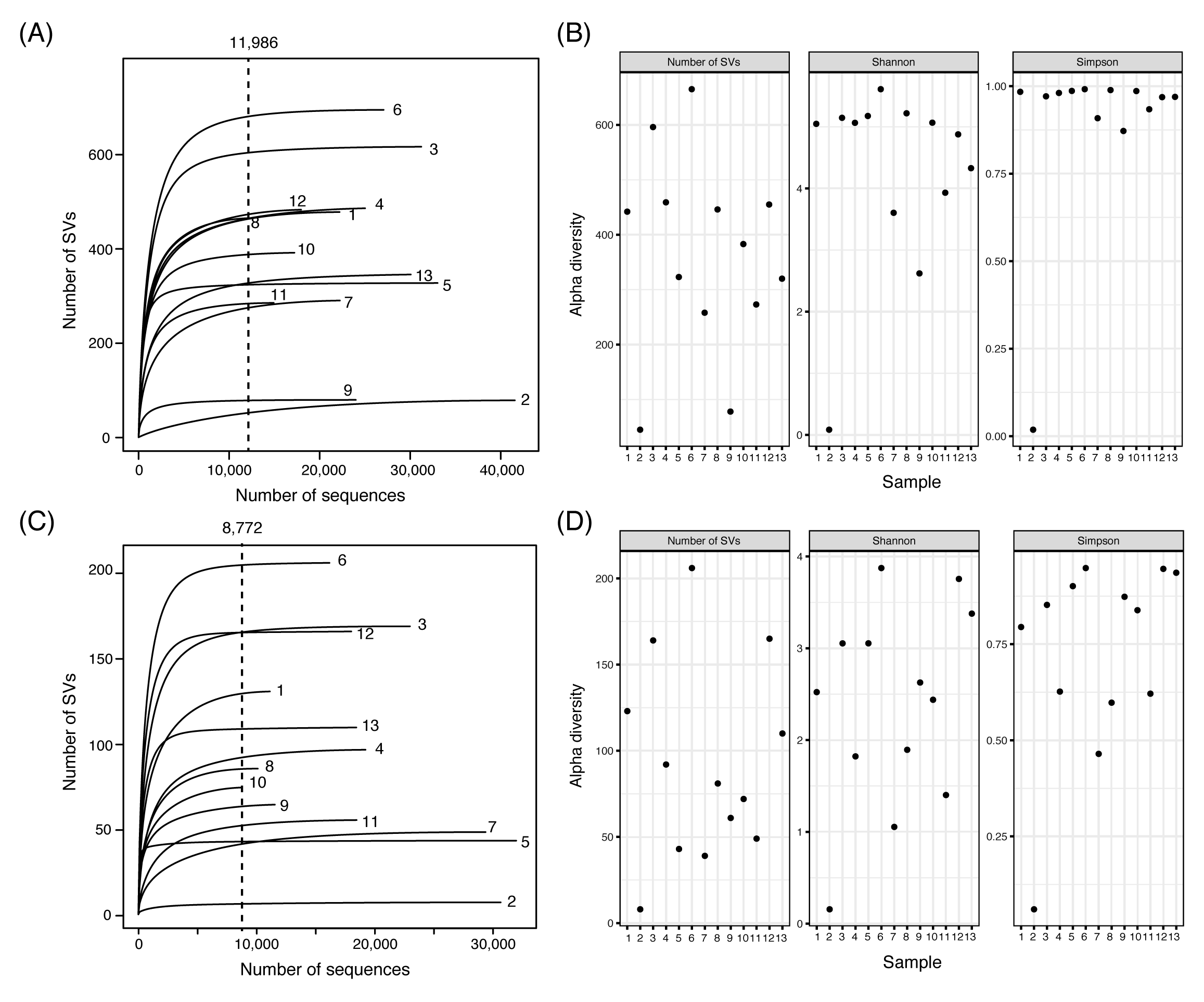


**S1 Fig. Alpha-diversity analyses.** Rarefaction curves of identified SVs in 16S (A) and 18S rRNA(C) gene analyses. Alpha diversity (numbers of identified SVs, Shannon index, and Simpson index) was estimated after rarefaction to 11,986 and 8,772 sequences for 16S (B) and 18S (D) rRNA genes, respectively.
